# Constrained optimization: evaluating possible packages of community health interventions with competing resource requirements in Galmudug, Somalia

**DOI:** 10.1093/heapol/czaf014

**Published:** 2025-03-11

**Authors:** Robert J Montgomery, Elaine Scudder, Caitlin Tulloch, Muna Jama, Naoko Kozuki, Baris Ata

**Affiliations:** Department of Operations Management, University of Chicago Booth School of Business, 5807 S. Woodlawn Ave., Chicago, IL 60637, USA; Research, Evaluation & Learning Department, International Rescue Committee, 122 E 42nd Street, New York, NY 10168, USA; Crisis Response, Recovery and Development Department, TechEx, and Health Department, International Rescue Committee, 122 E 42nd Street, New York, NY 10168, USA; Research, Evaluation & Learning Department, International Rescue Committee, 122 E 42nd Street, New York, NY 10168, USA; Somalia Program, International Rescue Committee, Airport Road, Mogadishu, Somalia; Research, Evaluation & Learning Department, International Rescue Committee, 122 E 42nd Street, New York, NY 10168, USA; Department of Operations Management, University of Chicago Booth School of Business, 5807 S. Woodlawn Ave., Chicago, IL 60637, USA

**Keywords:** resource allocation, maternal and child health, infant mortality, maternal mortality, decision-making, cost-effectiveness analysis, community care

## Abstract

Investment in community health worker (CHW) programs has allowed health systems to reach previously underserved rural and remote populations. As a result, CHWs are often burdened with responsibilities to deliver large packages of services, at times without sufficient human, financial, or health resources. To design a community-level program that saves maternal and newborn lives while operating within resource limitations, we used constrained optimization (a mathematical process for finding the solution to a stated objective while accounting for listed requirements) to construct a model for select villages in Galmudug State, Somalia. After establishing the resource requirements for delivering 25 evidence-based maternal and neonatal interventions, we used the Lives Saved Tool and optimization techniques to determine the package of care that leads to the most projected lives saved. With a cadre of 1450 female health workers and a budget of $435 000 for maternal and neonatal health commodities and programming over 1 year, we calculated that the optimized set of interventions for Galmudug could avert 15% of the 4132 projected maternal and neonatal deaths in 2024. We also conducted sensitivity analyses to show how the optimal combination of interventions and the number of lives saved change as the resource levels change. The model provides practitioners with a new tool and accompanying approach to evaluate possible packages of community health interventions with competing resource requirements.

Key messages

- Constrained optimization is a useful tool in designing or evaluating the health interventions comprising a package of care to offer in community health programs, by ensuring the resources are used in the most effective way to achieve a defined objective.
- Given the resources available for a maternal and newborn health program in Galmudug, Somalia, we estimate that the set of interventions selected by the model we provide can avert 15% of the annual maternal and neonatal deaths.
- Due to the model’s selection of several low-cost interventions, our sensitivity analysis indicates that recruiting more health workers leads to more lives saved than increasing funding for health commodities.

## Introduction

The maternal and newborn health (MNH) community is at a critical juncture—progress toward reductions in maternal and newborn mortality has stagnated, and global objectives will only be achieved if efforts to address the leading causes of death are prioritized and resourced within conflict-affected and fragile settings. Globally, 58% of maternal deaths, 37% of newborn deaths, and 36% of stillbirths occur in countries with 2024 appeals for international humanitarian assistance, highlighting the need to focus on improving MNH in these contexts (Align 2024). Furthermore, progress depends, in part, on programs extending beyond the easy-to-reach portions of the population to those without well-resourced facilities and requiring community-level care.

Somalia, in particular, is at the forefront of these concerns, with continued health and humanitarian crises caused by and resulting in conflict, instability, and poverty, along with uncertainty from the growing threat of changing climate conditions (Directorate of National Statistics, Federal Government of Somalia 2020). These factors threaten to imperil the already lacking maternal and neonatal health services provided within the country. Somalia has among the world’s highest rates of maternal mortality, with 692 deaths per 100 000 live births (Aweis et al. 2024). Neonatal mortality also ranks in the top five globally with ∼37 newborn deaths for every 1000 births, >80% of which are due to prematurity, asphyxia, complications during birth, or infections (UNICEF 2019, 2024). The country overall is severely under-resourced, with only four doctors, nurses, or midwives for every 10 000 people (Rizwan 2018). In resource-constrained settings like Somalia, community-based maternal and newborn care can help expand access to life-saving services and improve maternal and newborn survival, especially if done so in a way where these limited resources are optimized.

Constrained optimization—the process of minimizing or maximizing a function to arrive at a solution that satisfies a defined set of resource constraints—is a common tool in operations research, which is increasingly used in public health applications (Crown et al. 2018, Varghese et al. 2020, Schmitt et al. 2021, Mohan et al. 2022). Using this tool, we aimed to develop a setting-specific, context-adaptable model to select the package of community-based MNH (CBMNH) interventions that would maximize the number of lives saved while accounting for the resources available in a specific setting, including the funding provided and the amount of time health workers have to provide these interventions.

Although the constrained optimization model is intended to be flexible in its application to different settings, we first formulated it for the female health worker (FHW) cadre in Somalia. The program was established by the Somalia Ministry of Health (MOH) and funded by the World Bank to deliver essential health services at the household and community levels (Jama et al. 2023). While not yet widespread, the country intends to scale up the FHW cadre over the coming years. Thus, the authors’ organization saw value in engaging stakeholders through a contextualized process to ensure the resources available for this cadre are used to achieve the greatest impact. The authors’ organization partnered with the MOH, state government officials, professional associations, and partner implementing agencies to define and ultimately pilot a package of MNH interventions to be delivered by FHWs in Galmudug, Somalia. Engaging national- and state-level partners allowed for sourcing first-hand information on the practical constraints that often hamper the delivery of CBMNH services—particularly health worker time, training capacity, and the policy environment (including commodity availability). By incorporating stakeholder input throughout the modeling process, we aimed to both improve the accuracy of the model and develop trust in the resulting recommendations.

The FHW cadre used in this model is located in Galmudug, Somalia, which is in the center of the country and contains the city of Dhuusamareeb. The state is comprised of the Galgaduud and Mudug regions. The Somali Health and Demographic Survey- Galmudug Report provides an overview of the health and economic conditions within the state (Somalia National Bureau of Statistics (Formerly Directorate of National Statistics, Federal Government of Somalia) 2021). About 31% of Galmudug’s population are nomadic and primarily depend on livestock for income. The state shares similar health challenges with the country as a whole, including a shortage of qualified health professionals and insufficient financial resources. The state also experiences frequent droughts, further complicating health service delivery and economic opportunities.

Despite the focus of this paper on the FHW cadre in Somalia, the model provided is sufficiently general so as to be applicable to community health worker (CHW) cadres in other settings. An expansive evidence base has demonstrated the effectiveness of CHW cadres in improving care-seeking, health literacy, and health behaviors in numerous contexts, which has led to an increased reliance on them to perform an ever-expanding list of tasks (Perry et al. 2014). Recognizing the potential of these cadres has also led many countries to set ambitious targets for providing comprehensive care at the community level. In contexts where health systems are stressed by low resources, conflict, or fragility, these aims can be difficult to achieve. Studies on CHW programs regularly cite the difficulty of recruiting and retaining qualified CHWs as a primary reason why community health programs fail to meet their objectives (Tshering et al. 2018). The widespread issue of over-tasking CHWs, especially those who are unpaid volunteers, is a central finding of the review by Ballard et al. (2023), which recommends that “health providers should assess community health workers’ workloads a priori and measure actual time use” In addition to the critical task of increasing the human and material resources available for CHW programs, we believe that improvements can also be achieved by reassessing the process by which interventions are selected for inclusion in community health packages.

## Materials and methods

Constrained optimization, like most modeling approaches, involves translating a problem into mathematical language so that the tools available to analyze mathematical structures can be applied to solve or better understand the problem. Although the constraints used in constrained optimization problems are often presented in their mathematical form, they represent clear statements that can be expressed in words. Constructing an optimization problem in this way involves specifying requirements for an acceptable solution to the problem. In addition to solution requirements, the objective of the problem (e.g. maximize the number of maternal and child deaths prevented) must be defined in the form of a function. To inform a decision-making process, certain variables must be subject to the decision-maker’s discretion. These variables are labeled “decision variables,” and their values are permitted to change as part of the optimization processes. A solution to the problem is thus presented as a feasible set of values for each of these decision variables. This is in contrast to the model parameters, which are the values that are defined according to the setting where the model is applied. For instance, the decision of whether or not to offer chlorhexidine for clean cord care as part of the package of care is a decision variable, whereas the number of births in a year and the efficacy of chlorhexidine in preventing sepsis are examples of model parameters. Once the objective function, model parameters, decision variables, and constraints are defined, the process of finding a solution that is optimal according to the problem definition, or “close enough” to optimal to satisfy stated tolerances, relies on optimization techniques and algorithms. For the interested reader, we provide additional information on constrained optimization applied to other health settings in Supplementary Appendix A, as well as the specific optimization techniques used for this paper in Supplementary Appendix B.

We chose to approach this problem using constrained optimization as it accomplishes four aims: it allows us to provide structure to the evaluation process, consider multiple competing resources, set a defined objective, and replicate the approach in other settings. In doing so, we are attempting not to favor any specific health interventions but to offer a fair assessment of what is realistically achievable and which combinations of health interventions lead to the most projected lives saved in a given situation. We discuss the strengths and limitations of this approach further in the Discussion section.

We now present the model in its general form. We begin by describing each component of the lives-saved calculation as defined in the Lives Saved Tool (LiST) (Walker et al. 2013). LiST is a mathematical model, supported by a collection of country-level data, that allows users to estimate the impact on mortality from changes to the coverage levels of various health interventions for any of the countries within the database. After establishing the components from LiST used in our model, we write out the mathematical formulation comprising the model. We then explain each of the constraints.

Our model has two sets of decision variables: the first set denotes the incremental coverage level for different interventions, while the second set indicates whether or not each intervention is selected for implementation. To be specific, decision variable ${x_j}$ represents the change in coverage (i.e. the selected percentage of population coverage minus the current percentage of population coverage) of intervention *j* for $j = 1, \ldots ,J$, where *J* is the number of interventions under consideration. Decision variable ${y_j}$ is binary and set to 1 if intervention *j* is selected (i.e. identified by the solved model as being included in an optimal package of services), and 0 otherwise. We let $x = \left( {{x_j}} \right)$ and $y = \left( {{y_j}} \right)$ denote the vectors of decision variables for notational convenience.

Using these decision variables, we can calculate the number of projected lives saved using the parameters shown in Table 1. These values, in addition to others used to populate the model, are provided on a country-level basis in LiST. We detail the tool itself in Supplementary Appendix C, where we offer additional references.

**Table 1.** Parameters included in the model on a country-level basis

*c_j_*	Current level of coverage for intervention *j*
*D_N_*	Annual number of neonatal deaths
*D_M_*	Annual number of maternal deaths
*r_i_*	Percentage of deaths attributed to cause of death *i*
*f_ij_*	Affected fraction for cause of death *i* and intervention *j*
*e_ij_*	Efficacy for cause of death *i* and intervention *j*
*u_j_*	Level of coverage which equates to full coverage for intervention *j*
*d*	Available increase in coverage determined by the number of workers available
*α_j_*	Annual number of commodities required to reach full coverage of intervention *j*
*β_j_*	Annual commodities available for intervention *j*

The resulting mixed-integer program for maternal and newborn community healthcare optimization is given as follows:


(1) $$\begin{aligned}\mathop {max}\limits_{x,y} \,\, & {D_N}\mathop \sum \limits_{i = 1}^8 {r_i}\left[ {1 - \mathop \prod \limits_{j = 1}^J \left( {1 - {f_{ij}}\frac{{{e_{ij}}{x_j}}}{{1 - {e_{ij}}{c_j}}}} \right)} \right] + {D_M}\mathop \sum \limits_{i = 9}^{17} {r_i} \\ & \nonumber \left[ {1 - \mathop \prod \limits_{j = 1}^J \left( {1 - {f_{ij}}\frac{{{e_{ij}}{x_j}}}{{1 - {e_{ij}}{c_j}}}} \right)} \right]\end{aligned}$$

subject to


(2) $$Ax \le a$$

(3) $$By \le b$$

(4) $${\mathrm x}_{\mathrm j}\leq\min\left({\mathrm u}_{\mathrm j}-{\mathrm c}_{\mathrm j},\mathrm d,\beta_{\mathrm j}/\alpha_{\mathrm j}\right),\,\,\,\,\,\,\,\,\,\forall\;\mathrm j$$

(5) $${\mathrm x}_{\mathrm j}\leq{\mathrm y}_{\mathrm j},\,\,\,\,\,\,\,\,\,\,\,\,\,\,\,\,\,\,\,\,\,\,\,\,\,\,\,\,\,\,\,\,\,\,\,\,\,\,\,\,\,\,\,\,\,\,\,\,\,\,\,\,\,\,\,\,\,\,\forall\;\mathrm j$$

(6) $${\mathrm y}_{\mathrm j}\in\left\{0,1\right\},\,\,\,\,\,\,\,\,\,\,\,\,\,\,\,\,\,\,\,\,\,\,\,\,\,\;\,\,\,\,\;\;\,\,\,\,\,\,\,\,\,\,\,\,\,\,\forall\;\mathrm j$$

(7) $$x_j\geq0,\,\,\,\,\,\,\,\,\,\,\,\,\,\,\,\,\,\,\,\,\,\,\,\,\,\;\;\,\,\,\,\,\,\,\,\,\,\,\,\,\,\,\,\,\,\,\,\,\,\,\,\,\,\,\,\,\,\,\forall\;\mathrm j$$

Equation (1) is comprised of two expressions: the first accounts for the eight neonatal causes of death, signified by the sum from $i = 1$ through 8; the second accounts for the nine maternal causes of death, signified by the sum from $i = 9$ through 17. To arrive at Equation (1), the model emulates the LiST approach by using the equations provided in one of the LiST supporting documents (Winfrey et al. 2011).

Winfrey et al. (2011) describe the methods for how the LiST estimates “the impact of scaling up interventions on neonatal and child mortality.” As the objective function of the optimization model needs to be written using a single equation, it combines Equations (1) and (2) from Winfrey et al. (2011). Table 2 provides the notation used in Winfrey et al. (2011) which, for simplicity and brevity, is altered in the optimization model.

**Table 2.** Parameters and variables included in the LiST lives saved equation

*R_i,j,a,t_*	Cause of death *j* mortality reduction for age band *a* from intervention *i* at time *t*
*I_i,j,a_*	Effectiveness of intervention *i* on cause of death *j* for children in age band *a*
*C_i,a,_*_0_	Coverage of intervention *i* in age band *a* at time 0 (before changing coverage)
*C_i,a,t_*	Coverage of intervention *i* in age band *a* at time *t*
AF*_i,j,a_*	Affected fraction of intervention *i* on cause of death *j* for children in age band *a*

Equation (8), taken directly from Winfrey et al. (2011), provides the equation for the proportional reduction in mortality from changes in coverage for an individual intervention:


(8) $${R_{i,j,a,t}} = \left[ {{I_{i,j,a}} \times \left( {{C_{i,a,t}} - {C_{i,a,0}}} \right)/\left( {1 - {I_{i,j,a}} \times {C_{i,a,0}}} \right)} \right] \times {\mathrm{A}}{{\mathrm{F}}_{i,j,a}}$$

The functional form of Equation (8) is justified in the following way. The numerator, ${I_{i,j,a}} \times \left( {{C_{i,a,t}} - {C_{i,a,0}}} \right) \times {\mathrm{A}}{{\mathrm{F}}_{i,j,a}}$, gives an estimate of the reduction in mortality from the increase in coverage. By multiplying the effectiveness of the intervention by the change in coverage and the affected fraction, the expression accounts for both the documented efficacy of the intervention and the specific underlying mechanisms for the cause of mortality that the intervention targets. This estimate of the reduction in mortality needs to be adjusted to reflect that there may be a portion of the population receiving treatment through current coverage levels already accounted for in the mortality rate. The denominator $\left( {1 - {I_{i,j,a}} \times {C_{i,a,0}}} \right)$ adjusts for these deaths already prevented, which appear in a reduced mortality rate. For example, a 10% increase in coverage for a specific intervention will result in the same numerator, i.e. estimate of the reduction in mortality, regardless of the current coverage level, assuming fixed effectiveness and affected fraction. However, if the current coverage is greater than zero, then the mortality rate, to which the reduction in mortality is applied to produce the number of lives saved, will reflect these effective treatments from current coverage levels and have a lower base. The adjustment then ensures that any 10% increase in coverage results in the same number of lives saved, as long as ${C_{i,a,t}} \le 1$, regardless of the current coverage level.

As an example case, Winfrey et al. (2011) use an increase in oral rehydration solution coverage to show how this equation is applied to a specific instance. As an additional example, this same process is shown here, using kangaroo mother care (KMC). First, assume that the current level of coverage for KMC is 5%. If the coverage level for the KMC intervention alone is increased to 15%, coupled with the effectiveness of 0.51 and the fraction of premature deaths that can be prevented by KMC of 0.58, then the calculation for the reduction in mortality is as follows:


$$\begin{aligned}{R_{i,j,a,t}} = & \left[ {0.51 \times \left( {0.15 - 0.05} \right)/\left( {1 - 0.51 \times 0.05} \right)} \right] \\ & \times 0.58 = 0.03.\end{aligned}$$

This result indicates that, under the set of parametric assumptions provided, 3% of neonatal premature deaths can be prevented by increasing KMC coverage from 5% to 15%. The annual number of neonatal deaths due to prematurity within the population is then needed to calculate the number of lives saved. Using Somalia, the annual number of neonatal deaths is ${D_N} = 26\,4\,13$ and the percentage of neonatal deaths attributed to prematurity is ${r_5} = 22.63\% $. The total number of neonatal deaths in this population attributed to prematurity is then 5977. Therefore, a 3% reduction in neonatal deaths due to prematurity is equivalent to $5977\times3\%=179\,$ lives saved.

When multiple interventions that target the same cause of mortality are scaled up, LiST sequentially applies these interventions so as to avoid double counting (Walker et al. 2013, p. 2). In Winfrey et al. (2011), the total impact of all scaled interventions on a specific cause of death is the product of each intervention on the remaining mortality and is written in the following manner:


$$\begin{aligned}{R_{j,a,t}} = & 1 - \left( {1 - {R_{1,j,a,t}}} \right) \times \left( {1 - {R_{2,j,a,t}}} \right) \times \left( {1 - {R_{3,j,a,t}}} \right) \\ & \times \left( {1 - {R_{4,j,a,t}}} \right) \ldots ,\end{aligned}$$

or more succinctly as,


(9) $${R_{j,a,t}} = 1 - \mathop \prod \limits_{i = 1}^I \left( {1 - {R_{i,j,a,t}}} \right).$$

Using the expression for ${R_{i,j,a,t}}$ in Equation (8) along with Equation (9) results in the full expression for the total impact of all interventions on a single cause of mortality for a specific age band as shown in Equation (10):


(10) $$\begin{aligned}{R_{j,a,t}} = & 1 - {\prod}_{i = 1}^I \left( 1 - \left[ {{I_{i,j,a}} \times \left( {{C_{i,a,t}} - {C_{i,a,0}}} \right)/\left( {1 - {I_{i,j,a}} \times {C_{i,a,0}}} \right)} \right]\right. \\ & \nonumber \left. \times {\mathrm{A}}{{\mathrm{F}}_{i,j,a}} \right)\end{aligned}$$

The constrained optimization model replaces ${I_{i,j,a}}$ with ${e_{ij}}$ as the efficacy for cause of death *i* and intervention *j*, substitutes ${C_{i,a,t}} - {C_{i,a,0}}$ with ${x_j}$ as the change in coverage level for intervention *j*, replaces ${C_{i,a,0}}$ with ${c_j}$ as the current level of coverage for intervention *j*, and replaces ${\mathrm{A}}{{\mathrm{F}}_{i,j,a}}$ with ${f_{ij}}$ as the affected fraction for cause of death *i* and intervention *j*. These changes in notation result in Equation (11) (note that *j* indexes the interventions and that *i* indexes the causes of death which are opposite of the the indices used in the Winfrey et al. (2011) paper).


(11) $${R_{i,a,t}} = 1 - \mathop \prod \limits_{j = 1}^J \left( {1 - {f_{ij}}\frac{{{e_{ij}}{x_j}}}{{1 - {e_{ij}}{c_j}}}} \right)$$

To then calculate the reduction in deaths from all changes in intervention coverage, one can sum the number of lives saved for each cause of death *i*. The number of neonatal deaths calculated using population projections and the current mortality rates is ${D_N}$. ${r_i}$ is the percentage of neonatal deaths that can be attributed to cause of death *i*. The total neonatal deaths from cause of death *i* is then given as ${D_N}{r_i}$. Similarly, ${D_M}{r_i}$ gives the total number of maternal deaths from cause of death *i*. The total number of lives saved through changes to intervention coverage can then be estimated using the expression:


$$\mathop \sum \limits_{i = 1}^I {D_N}{r_i}{R_{i,a,t}} + \mathop \sum \limits_{i = 1}^I {D_N}{r_i}{R_{i,a,t}},$$

which serves as the equation underlying the model’s objective function.

Matrices A and B in Equations (2) and (3) are both $3 \times J$ dimensional consumption matrices; their columns correspond to different interventions. Likewise, a and b are 3-dimensional capacity vectors—these capture the relationship between the components necessary to implement CBMNH programs and the total resource budget for each of the components. In the descriptions of the constraints to follow, we let ${A_i}$ and ${B_i}$ denote the $i{\mathrm{th}}$ row of matrices A and B, respectively, for $i = 1,2,3$. And, let ${a_i}$ and ${b_i}$ denote the ${i^{th}}$ component of each vector.

- Delivery capacity: the constraint ${A_1}x \le {a_1}$ ensures the total time required for CHWs to deliver the level of coverage for the selected interventions does not exceed the available time to do so. The resource budget for delivery time, ${a_1}$, is a key factor to understand in program planning. It can be shifted upward or downward to reflect components like travel time, the target number of households each CHW serves, and the number of other health modules CHWs are tasked with delivering.
- Supervision capacity: the constraint ${A_2}x \le {a_2}$ ensures the supervisors’ time needed to properly oversee the interventions requiring in-service training does not exceed the available time to provide supervision to CHWs. Particularly where CHWs may be asked to provide new and technically complex interventions, the availability of supervisors to ensure properly delivered quality care can be a limiting factor.
- Commodity scaling coverage cost: the constraint ${A_3}x \le {a_3}$ ensures the total cost to increase intervention coverage, based on the price of commodities necessary for different interventions, is less than or equal to the budget available to scale up interventions.
- Curriculum time: the constraint ${B_1}y \le {b_1}$ ensures that the curriculum time required to train interventions does not exceed the curriculum time available for maternal and neonatal care. This constraint is especially important when new cadres of CHWs are hired, as the feasible time for training can limit the effective roll-out of new components of a service package.
- Intervention start-up cost: the constraint ${B_2}y \le {b_2}$ ensures that the total cost to establish new interventions does not exceed the budget available for that purpose. These costs can include, but are not limited to, those necessary to establish a supply chain for a medication not currently available at scale within the country, or those associated with policy change processes for the delivery of a new intervention at the community level.
- Permission to offer at the community level: the constraint ${B_3}y \le {b_3}\,$ ensures that an intervention can only be included in the recommended package if it is permitted for delivery at the community level by the country’s government and policies. Some interventions, such as oral antibiotics for sepsis or misoprostol for postpartum hemorrhage, have been shown to be feasible for community-level delivery, but concerns about CHW’s capacity to deliver the interventions or properly control the distribution of the associated commodities mean that the feasible set of interventions will vary between countries.

Equation (4) ensures that the change in coverage for intervention *j* does not exceed a practically feasible level. We define this level as the minimum of either: one, full coverage (${u_j}$) minus the current coverage of the intervention (${c_j}$); two, the maximum available coverage (*d*) determined by the size of the CHW cadres; or three, the maximum achievable coverage for intervention *j* if there are a limited number of available medications or health commodities required for delivering intervention *j*. Equation (5) states that the coverage level of intervention *j* (${x_j}$) can only be increased if the intervention is selected (i.e. if ${y_j} = 1$). Equations (6) and (7) ensure ${y_j}$ is binary and ${x_j}$ is nonnegative, respectively, for $j = 1, \ldots ,\,J$.

To properly measure the resources used for certain constraints, we need to translate the level of coverage for each intervention into the equivalent number of treatments needed to achieve that increase in coverage. As the individuals identified as requiring treatment will not be the same for all interventions (for instance, iron fortification is administered to pregnant women but not newborns, infants, or children), we match each intervention with a target population. A target population is a subset of the country’s population defined by a common feature, e.g. pregnant women. The sizes of relevant target populations are provided by LiST for each country. Examples of these target populations can be found in Table 10 of Supplementary Appendix D. The number of treatments needed to achieve full coverage for an intervention is calculated by multiplying the target population by the percentage of the target population requiring that intervention. Multiplying the number of treatments required to achieve full coverage of intervention *j* by the change in coverage (${x_j}$) provides the number of treatments required for that change in coverage.

Having established the components of the general model, we can now apply the analysis to specific settings, beginning with the CBMNH program in Somalia. In Somalia, the FHW cadre provides healthcare to both rural and urban populations, either supplementing the services provided at health facilities or serving as the main point of contact for populations with limited access to health facilities. FHWs are health promoters who receive training to counsel, diagnose, and treat conditions across various modules, including MNH.

The model aims and parameters were sourced during a 3-day stakeholder workshop in Mogadishu, Somalia in October 2022, bringing together state-, national-, and global-level actors. The workshop included an introduction to constrained optimization, detailed discussions about the feasibility and resource requirements for each intervention, and planning for how the resulting model insights would be used in a pilot program. Critically, this workshop allowed us to source information on key resource constraints (e.g. the time FHWs have available for MNH delivery, the time it would take to train FHWs on each newly introduced service, and the costs of procuring commodities) directly from health actors who live and work in Somalia.

LiST includes 33 interventions that directly or indirectly affect either maternal or neonatal mortality or both. We relied on input from technical advisors with expertise in MNH to narrow the list down to 25 interventions that can reasonably be delivered at the community level in a stable setting with a strong health system. To underscore the need for optimization techniques in solving this problem, if all 25 interventions were evaluated only for the *y* decision variables of including or not including each intervention in the package, then there are ${2^{25}}$ (or 33 554 432) possible combinations to consider. Within Somalia’s health program, 16 of these 25 interventions were further deemed infeasible to provide at the community level—this is captured in the constraint for ${B_3}$ of Equation (3). The interventions included in the model, their current levels of coverage in Somalia according to LiST, and their statuses as either feasible or infeasible for community delivery in Somalia are shown in Table 3.

**Table 3.** MNH intervention current coverage levels and community level feasibility in Somalia

Intervention	Current coverage (%)	Feasible at the community level
Tetanus toxoid vaccination	67	No
Multiple micronutrient supplementation in pregnancy	0	No
Iron supplementation in pregnancy	0	Yes
Calcium supplementation	0	Yes
Balanced energy supplementation	0	No
KMC	5	Yes
Breastfeeding promotion	9	Yes
Handwashing with soap	10	Yes
Prevention of malaria in pregnancy	2	Yes
Basic sanitation	38	Yes
Clean birth environment	7	No
Clean cord care	8	Yes
Immediate drying and additional stimulation	8	No
Neonatal resuscitation	4	No
Uterotonics for postpartum hemorrhage	7	No
Syphilis detection and treatment	4	No
Thermal regulation	9	Yes
Manual removal of placenta	3	No
Point-of-use filtered water	4	No
Malaria case management	1	No
Injectable antibiotics for neonatal sepsis	9	No
Safe abortion services	3	No
Oral antibiotics for neonatal sepsis	0	No
Postabortion case management	0	No
Antibiotics for treatment of dysentery	0	No

We use the Somalia neonatal mortality rate of 36.8 deaths per 1000 live births, which is provided in LiST. For the maternal mortality ratio, in lieu of the rate of 621 deaths per 100 000 live births provided in LiST, we used the ratio provided by the MOH of 692 deaths per 100 000 live births. As a baseline for calculating the number of neonatal and maternal deaths (${D_N}$ and ${D_M}$), we use the 2023 projected number of births in Somalia, which is 716 518 according to LiST. Together, these numbers provide a projection of 26 413 neonatal deaths and 4958 maternal deaths in 2023 across the entire country.

Tables 11 and 12 of Supplementary Appendix D list the various causes of death, along with their respective contributions to predicted neonatal and maternal mortality in Somalia. The proportions of neonatal deaths attributed to each cause are from 2017 and sourced from the WHO estimates between 2000 and 2017 (WHO, 2017). The proportions of maternal deaths attributed to each cause are from 2013 (Say et al. 2014).

The final sets of parameters needed for the objective function of the model are the Affected fraction (${f_{ij}}$) and Efficacy (${e_{ij}}$) values. With 17 causes of death and 25 interventions, we list these values in two $17 \times 25$ matrices. We use the values from the LiST database for these parameters, except for a select number, which we adjust to reflect the estimates provided by the public health experts who participated in the aforementioned workshop. The matrices for Affected fraction and Efficacy values are provided in Supplementary Appendix D. Several interventions only affect one cause of death—for instance, iron supplementation in pregnancy is efficacious only against deaths resulting from congenital anomalies in neonates. The other interventions affect at most four causes of death.

While the model can project the impact of coverage for up to 100% of households, the actual reach of these interventions is limited by the number of FHWs available for CBMNH programming. The capacity for delivering health interventions is in turn set by the feasible increase in coverage (*d*). For the Somalia setting, we assume the availability of 1450 FHWs, who are each responsible for roughly 200 households, as prescribed by country-level policy, with an average of six people per household. These assumptions allow FHWs to reach 1.74 million of the 17.6 million people in Somalia or 9.88% of the population. Thus, the model does not allow total coverage for each intervention to exceed current coverage plus $d = 9.88\% $. The maximum total coverage levels for each intervention are listed in Table 17 of Supplementary Appendix D. There are plans within Somalia to increase the number of FHWs over the coming years. In order to understand how this change will alter the optimal package of interventions and the number of lives saved, we perform sensitivity analysis in the Results section.

To calculate the number of treatments needed to achieve full population coverage, we identify six population groups that are eligible for different interventions (see Table 10 of Supplementary Appendix D). In Table 18 of Supplementary Appendix D, we pair each intervention with its target population and provide estimates for the percentage of the target population meeting the criteria for each intervention.

The values for the resource constraints of the three-dimensional vectors a and b are shown in Table 4. The detailed calculations for these values are included in Supplementary Appendix G. Due to the limited number and nature of the interventions allowable in the Somalia setting (Table 3), we have decided to remove the constraint regarding the cost to establish new interventions, as many of the interventions under consideration are already established, to some degree, within the country. Instead, we focus on the cost of commodities necessary to scale coverage. Similarly, we would ordinarily provide the annual number of commodities attainable and the commodities required for full coverage for each intervention in Somalia, as included in Equation (4). However, we do not have exact numbers for these values at this time. Instead, we elect to use a binary constraint for whether or not the commodities for each intervention were deemed available during consultations. If these values do become available, the constraint can easily be adjusted to reflect this update.

**Table 4.** Constraints on FHWs

Constraint	Budget
(*a*_1_) Total time available per month for FHWs to perform MNH care	22 602 h
(*a*_2_) Total supervisor time per month available to oversee FHW in-service training	3927 h
(*a*_3_) Total commodity budget available for scaling interventions	$435 000
(*b*_1_) Total time available in FHW Maternal and Neonatal curriculum	20 h
(*b*_2_) Total budget available for establishing new interventions	–
Allowable increase of intervention based on the number of FHWs	9.88%

## Results

The optimization model, using the identified Somalia health parameters and resource constraints, yields a set of eight suggested interventions to maximize the number of maternal and neonatal lives saved (Table 5). The package of care is projected to avert 612 maternal and neonatal deaths, which would account for 15% of the projected maternal and neonatal deaths in Galmudug, Somalia, in 2023.

**Table 5.** Optimal package of interventions for the Somalia setting

1.	KMC
2.	Clean cord care
3.	Thermal regulation
4.	Prevention of malaria in pregnancy
5.	Breastfeeding promotion
6.	Basic sanitation
7.	Handwashing with soap
8.	Iron supplementation in pregnancy

Of the 25 interventions identified as viable to offer at the community level for MNH care, only nine are under consideration within the Somalia program (Table 3). The reasons for excluding the majority of interventions range from logistical to cultural and were determined during the stakeholder workshop in Mogadishu. Policy feasibility is thus a major determinant of the optimal package of services in Somalia.

The model is a mixed-integer nonlinear program. We express the model in AMPL and use the KNitro solver to arrive at an optimal solution (for more detail, see Supplementary Appendix B). As we hope there will be interest in adapting this modeling exercise to other settings, we plan to make this model available for use and welcome inquiries about the results of the model using other sets of parameters. The model results are displayed in Table 6.

**Table 6.** Optimal package of interventions for the Somalia setting with 1450 FHWs

Intervention	*y_j_*	Coverage change, *x_j_* (%)	Coverage target (%)	Current coverage (%)
Tetanus toxoid vaccination	0		67	67
Multiple micronutrient supplementation in pregnancy	0		0	0
Iron supplementation in pregnancy	1	4.15	4.15	0
Calcium supplementation	0		0	0
Balanced energy supplementation	0		0	0
KMC	1	9.88	14.88	5
Breastfeeding promotion	1	9.88	18.88	9
Handwashing with soap	1	9.88	19.88	10
Prevention of malaria in pregnancy	1	9.88	11.88	2
Basic sanitation	1	9.88	47.88	38
Clean birth environment	0		7	7
Clean cord care	1	9.88	17.88	8
Immediate drying and stimulation	0		8	8
Neonatal resuscitation	0		4	4
Uterotonics for postpartum hemorrhage	0		7	7
Syphilis detection and treatment	0		4	4
Thermal regulation	1	9.88	18.88	9
Manual removal of placenta	0		3	3
Point-of-use filtered water	0		4	4
Malaria case management	0		1	1
Injectable antibiotics for neonatal sepsis	0		9	9
Safe abortion services	0		3	3
Oral antibiotics for neonatal sepsis	0		0	0
Postabortion case management	0		0	0
Antibiotics for treatment of dysentery	0		0	0

Within the optimal set of interventions, iron supplementation is limited to only a 4.15% increase because of its relatively high cost per treatment. Likewise, calcium has a relatively high cost per treatment and is excluded from the optimal set of interventions by the model. All other permissible interventions are increased by 9.88%, which is the maximum allowable increase given the number of FHWs (*d*). The only binding constraint under these conditions is the Commodity Scaling Coverage Cost. The amounts of each constrained resource used in the model solution are listed in Table 7.

**Table 7.** Constraint budget usage for the Somalia program

Constraint	Budget	Usage
Total time available per month for care	22 602 h	13 588 h
Total supervisor time per month for training	3927 h	713 h
Total commodity budget available for scaling interventions	$435 000	$435 000
Total time available in curriculum	1200 min	800 min

As previously mentioned, the results in Table 6 are heavily influenced by national-level policies regarding MNH and community-level service delivery. With this in mind, the model results help confirm which interventions can be provided in the short term using the available resources. However, the specific requirements for the community-based MNH programs in Somalia mean that these results are unlikely to generalize, except in other countries with similar policy constraints. To better understand the extent to which the policy constraints determine the results of the model, we relax the “permission to offer at the community level” constraint in Equation (3), with the exception of the two interventions related to abortion services, as the procedure is illegal in Somalia, as well as manual removal of the placenta, as the MOH wants to encourage all births to take place at health facilities. We then reran the model with the Somalia parameters. The results from relaxing most of the policy restrictions, while keeping all other constraints and parameters as before, are shown in Table 19 of Supplementary Appendix E.

The objective function of this solution has a value of 1990 projected lives saved or 48% of maternal and neonatal deaths in Galmudug state in 2023. This is roughly three times as many lives saved as the 612 in Table 6. The exchange of interventions between the two versions of the model is shown in Table 8. This expanded version maintains the budget to scale interventions as a binding constraint, while also exhausting the curriculum time available, making it a binding constraint as well. The resources used in this solution are shown in Table 20 of Supplementary Appendix E. The estimated increase in lives saved can help determine how to prioritize future investments and policy efforts—for example, presently unavailable interventions could be ranked based on their contributions to the additional lives saved, and the most life-saving interventions given further consideration for permission and feasibility.

**Table 8.** Changes to the optimal package of expanded interventions for the Somalia setting

Removed	Added
Breastfeeding promotion	Tetanus toxoid vaccination
Handwashing with soap	Multiple micronutrient supplementation in pregnancy
Basic sanitation	Balanced energy supplementation
	Clean birth environment
	Immediate drying and stimulation
	Injectable antibiotics for neonatal sepsis
	Oral antibiotics for neonatal sepsis

**Table:** 

Participant	Position at the time of the workshop*j*
Dr Hawa Abdi	SORDI
Dr Mohamed Abdirahman	Acting Child Health Manager, MoH
Halimasa’dia Abdi Ali	Community Health Manager, IRC
Dr Hodan Ali	Family planning focal point, MoH
Deka Mohamed Nor Aliyow	RH Officer, MoH
Abdihakim Mohamed Diriye	Director of Policy and Planning
Dr Ubah Farah	Director of the Family Health Department
Deka Abubakar Gaal	UNFPA
Dr Ibrahim Guled	Secretary General
Dr Muna Jama	IRC Research Project Coordinator
Dr Mohamed Jimale	SORDI
Abdijamal Mire	IRC Dhusamareb Field Coordinator
Abdisalaam Abdullahi Mohamud	Director Community Health
Abukar Mohamud	IRC Deputy Director of Programs
Mustafa Mohamed	Somalia Community Health Strategy Focal Point, MoH
Ridwaan Hassan Abdi	UNFPA
Yusuf Omar	Head of Supply Chain
Elaine Scudder	IRC Maternal & Newborn Health Advisor
Dr Yahye Shoole	Director
Caitlin Tulloch	IRC Director of Best Use of Resources
Dr Mohamud Abdi Yunis	Senior Reproductive Health Manager, IRC

As demonstrated from the results in Table 8, adjustments can be made to the base setting to test how the optimal policy responds to changes in the operating constraints or parametric assumptions. We conduct a similar exercise in Supplementary Appendix E, where we provide a relaxed version of the permitted interventions, which includes the additional interventions but omits those interventions that require injections. While relaxing the policy constraints is an interesting hypothetical exercise, in many settings the interventions requiring injections are beyond the skill set or comfort level of CHWs and can require a sophisticated supply chain not available in all settings. Removing these interventions from consideration thus aligns the policies more closely with what may be feasible in most settings. This version of the model results in 1335 lives saved, which represents a more than doubling of the number of lives saved over the projection from the original model.

A reasonable approach to constructing a CBMNH program is to rank interventions by their cost-effectiveness ratios and prioritize the most cost-effective interventions. As a point of comparison, we consider the interventions included in an analysis of the cost-effectiveness of maternal and neonatal health interventions in Ethiopia (Memirie et al. 2019). Of the 13 interventions in their study, 6 were considered in the relaxed restriction version of the Somalia model; these included (in order of their USD/DALY-averted ranking): (i) neonatal resuscitation, (ii) KMC, (iii) injectable antibiotics for neonatal sepsis, (iv) tetanus toxoid (pregnant women), (v) syphilis detection and treatment (pregnant women), and (vi) calcium supplementation. Only three of these interventions were selected in our optimization process. The three that were left out were ranked 1st, 11th, and 13th in terms of cost-effectiveness. This highlights two main takeaways. First, the model performs its own cost-effectiveness calculation when applying the budgetary constraint, leading to the two most expensive interventions on a cost-effectiveness metric being left out of the optimal package. Second, there are other competing factors besides cost-effectiveness when considering the interventions to include in a package of care, which was a primary justification for the constrained optimization modeling exercise. This is clearly demonstrated by the most cost-efficient intervention, neonatal resuscitation, being left out of the optimal package. The parameters for neonatal resuscitation indicate that while inexpensive on a cost-per-DALY basis, the intervention requires a significant amount of time to train. It is left out of the optimal package because curriculum time is a binding constraint, and the model prioritizes teaching several interventions, which require less classroom time than neonatal resuscitation. Knowing this, it is reasonable to consider whether additional classroom time can be offered to teach this intervention or if this intervention should be taught selectively to FHWs who have demonstrated the ability to perform some of the more complex interventions.

To further understand the sensitivity of the results to the assumptions used to model the Somalia setting, we now show how the optimal package and the resulting number of lives saved change as key parameters change. Sensitivity analysis is one of the benefits of the constrained optimization approach, as, in addition to allowing for an improved understanding of how responsive the results are to certain values used in the model, it provides the ability to estimate the impact of possible future changes in the operating environment. We focus specifically on changes to the number of FHWs and the budget available, as they are parameters that are likely to change in Somalia. We extend the sensitivity analysis to include other parameters in Supplementary Appendix F.

**Figure 1.**
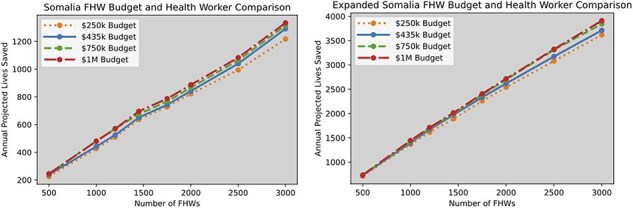
Projected lives saved for different combinations of FHW numbers and commodity budgets for the original Somalia setting (left) and the relaxed restriction version (right).

In Fig. 1, we consider eight different quantities of FHWs. We include the current goal for the number of FHWs in the program ($n = 1,450$) and consider a range from 500 to 3000 FHWs. We also consider four different annual commodity budgets of $250k, $435k (current budget), $750k, and $1 million. We provide the number of projected lives saved for each of these FHWs and budget combinations. In the left panel of Fig. 1, we solve the more restrictive original model, while in the right panel of Fig. 1, we consider the more permissive model from Table 19 of Supplementary Appendix E.

In Fig. 1, we see that the number of FHWs greatly impacts the number of lives saved. Interestingly, it is apparent from how closely the lines bunch together that increasing the commodities budget only has a small effect on the number of lives saved. This result underlines the significance of the parameter *d*, the maximum coverage achievable with the number of FHWs available, which grows linearly with the number of FHWs. These results suggest that under the current constraints and parameters for Somalia, interventions in the optimal package of care with little or no associated costs are some of the most efficacious in increasing the number of projected lives saved and that growing the number of FHWs through recruitment and retention should be prioritized over increasing the commodities budget.

## Discussion

The WHO currently provides a list of recommended interventions to offer as components of MNH programs (WHO, 2022). Additionally, resources such as LiST and cost efficacy analyses are available to aid in evaluating the impact of these interventions in specific country contexts. Despite the importance of these resources, we believe there still exists the need for a structured approach to assessing not just the potential impact of these interventions, but also the feasibility of offering combinations of these interventions in specific contexts. This is especially true when planning for community-level health programs, where issues of high turnover, overburdened health workers, limited infrastructure, and disrupted supply chains are known to exist. As such, our model attempts to bridge current gaps between planning and application by accounting for the resource requirements of the interventions under consideration and the availability of the necessary resources to deliver these interventions. Our use of constrained optimization further allows us to ensure the available resources are used most effectively.

The model presented in this paper is adaptable for several purposes. It can be used in the process of starting a new community-level MNH program within a country, as demonstrated in Galmudug, Somalia. If a community health program already exists in a given setting, it can serve as a means to assess the current services offered and identify potential changes to improve outcomes. Furthermore, it can function as a support tool to advocate for interventions not currently offered, specifically if the interventions are projected to lead to additional lives saved and the available resources are better used for those purposes. Additionally, if the program results are tracked after implementation, the model can serve as a reference to help understand where prior assumptions vary from the on-the-ground realities. In this way, it can help diagnose program issues and identify the sources of instances where outcomes may differ from expectations. For all of these purposes, the model provides a framework into which local expertise and perspectives can be readily incorporated.

As is especially important in resource-limited or aid-dependent contexts, the model allows us to test the impact of potential disruptions to the program, such as budget changes or the redirecting of resources (see the sensitivity analysis of the Results section). This is particularly valuable when health systems may find themselves facing frequent or unexpected shocks, including insecurity, infrastructure disruptions, supply chain challenges, public health emergencies or outbreaks, or redirecting or elimination of funds. When such a shock arises, a government, ministry, or agency could use this model to rapidly identify revisions to a community health program or package of care that reflects the new operating realities. To aid in this process, we can identify and prioritize interventions that persist in the model solution under a range of parametric changes, and favor these interventions so that the CBMNH program is still able to function at some capacity during disruptions.

As with any model, the quality of the results depends on the accuracy of the data used. We note that LiST uses national-level demographic surveys for all countries in their database and also stress that data collection in conflict-affected settings rarely, if ever, captures accurate representations of these settings. For our initiative in Somalia, subnational mortality or demographic data were unavailable, so we used the national-level survey data available in LiST. The accuracy of our model results, therefore, in part, depends on how closely the country-wide mortality and disease incidence rates approximate those of Galmudug, Somalia. Additionally, the inputs provided during the workshop were often estimates, and although they rely on information from those with intimate knowledge of the conditions, they are nonetheless subjective.

Because of the model’s reliance on LiST, our decision variables were selected from a set of interventions LiST identifies as contributing to the lives saved calculation. Notably, this criterion leaves out the routine service delivery systems through which these interventions are provided. For instance, many of these interventions can only be delivered within routine antenatal care (ANC) and prenatal care (PNC) visits. Those visits themselves and the relevant counseling provided during them are not specified in LiST as being lifesaving interventions and, therefore, are not identified as components of the optimal package of care by the model. Yet, it will be imperative that any policymakers using this model to design programs acknowledge routine service delivery platforms (like ANC and PNC visits) that offer care-seeking counseling, behavioral counseling, health literacy, and disease surveillance, among other services, are essential components of any such programs. Plainly, the model does not consider all aspects of a CBMNH program and focuses specifically on the resource requirements and mortality impacts of the specified interventions used as decision variables.

The model is currently designed with the objective of optimizing lives saved. Policymakers who wish to prioritize a different health outcome or metric can still use the model but would need to specify a new objective function to capture this focus. Such an extension would require an additional database that contains measurements for how increases in coverage for the interventions considered contribute to gains in the objective function. We previously noted one of the strengths of this modeling process, as the ability to track the program prescribed by the model after implementation. Although the continued evaluation of the model results is a useful exercise, it does require additional resources, including data tracking, which presents its own set of difficulties and may only be available in limited settings. It is thus at the discretion of the implementing group as to whether follow-up evaluations are the best use of funds, and these additional benefits may not be realized in all settings.

Although our model does consider other tasks that health workers need to perform by building them into the time budget to work on CBMNH care constraints (see Equation (26) of Supplementary Appendix G), it is still important to acknowledge that the amount of time available to provide these services can vary over time and between communities. Furthermore, the model assumes that all CHWs have an average level of experience and competency. Such simplifying assumptions are justified when trying to design a single-country program following a set curriculum. However, in practice, CHWs have a wide range of experience. We allude to this issue at the end of the Results section, where we suggest that those health workers who have shown the ability to perform more complex interventions could be trained in neonatal resuscitation despite the procedure requiring more training time than is available for most health workers. We emphasize that the model is intended as a starting point for program design and evaluation and stress the importance of context-specific considerations for its implementation.

Lastly, any type of modeling is limited and does not capture all the considerations in the setting where it will be applied. Furthermore, skepticism often abounds when attempting to apply mathematical modeling to established practices and systems. It is thus vital to connect users with the process from the start, not least because local interest-holders will be a critical source of information on resource constraints which are rarely captured elsewhere. Correctly framing the process and results as not telling those involved what to do, but instead providing guidance to aid in their decision-making is essential.

## Conclusion

In this paper, we provide a model for strategically selecting community health interventions in resource-constrained environments and use the Somalia FHW program in Galmudug as the model’s first application. We rely on LiST as a data source to calculate our objective of maximizing the number of maternal and neonatal lives saved and consulted with Somali experts to understand the resources necessary to deliver those interventions in that context. We use the model to identify the optimal combination of interventions under a set of constraints selected and adapted to the Somalia setting by authors of this paper, with significant input from practitioners within the Somali health system.

The package of CBMNH services that leads to the greatest number of projected lives saved in Somalia, given current constraints, includes eight of the nine interventions considered. The commodities budget available to scale up interventions is the binding constraint within the model. With a significant number of interventions excluded from the original analysis due to country-specific policy restrictions, we solve a version of the model with an expanded list of interventions to show the increase in the number of lives saved if additional interventions are considered. We also identify which interventions would be excluded from the original package of care to incorporate these new interventions. We finally show how the number of lives saved in Somalia is projected to change if the program budget and the number of FHWs change. This analysis suggests that increasing the number of FHWs is one of the best uses of resources for increasing the number of lives saved.

Relying on the work of our in-country partners, the program prescribed by our model is expected to launch in 2024. In addition, we have already performed a similar modeling exercise in South Sudan, using the same general model as our starting point. We believe that this type of analysis can play a valuable role in the program design and re-evaluation of community health programs.

## Supplementary Material

czaf014_Supp

## Reflexivity statement

There are four female and two male authors of this paper. Three authors are researchers, one in the field of Public Health and the other two in operations management (postdoc and professor). Another author is an economist working on the best use of resources for nonprofit aid organizations. The remaining two authors are maternal and neonatal health experts, one based in Washington, DC, with expertise in health programs in low-resource and conflict-affected states and the other a medical doctor based in Mogadishu, Somalia. We feel it is important to establish our rationale for publishing such a paper on a model that is intended to be country-driven and adaptable to various settings, recognizing the majority of the authors are US-based. The goal of this initiative was to provide local stakeholders with a tool to assist in project evaluation and not to prescribe a course of action. In this way, we hope that the tool will be used by the countries and not on behalf of them. However, the process of creating the global good itself—the mathematical model—was a partnership between the Booth School of Business at University of Chicago and the International Rescue Committee and engaged content experts, scientists, and academics over the course of several months to ensure the fidelity of the model and the reliability of the baseline data. In this regard, the authorship of this paper reflects the inputs required to generate the global good, but the authors anticipate that future applications and learnings of the model will be country-driven.

For example, in addition to the current ongoing pilot of the identified package in Somalia, a second similar effort/workshop was conducted in South Sudan, where the modeling exercise was used to verify that the country’s proposed package of MNH community care was feasible using the resources available. We anticipate findings of these implementation projects to be documented and disseminated by the local teams that are leading on those efforts. The authors intend to make themselves available for aiding other groups who wish to employ this model, or variations of it, within their own contexts, including offering model results reflecting stakeholder inputs, but we otherwise hope that this paper will encourage country stakeholders to see the potential in adapting this global good. We also flag that the purpose of the model is not to prescribe what services should be delivered, but to serve as a decision support tool, taking into consideration other contextual factors, including client demand, that may not be readily captured in a mathematical model.

We acknowledge that there are still dynamics at play between aid organizations and those receiving the aid. We hope the potential negative effects from these dynamics are lessened by the long-term relationships between the local stakeholders and in-country IRC offices, but are cognizant of their existence nonetheless.

This paper served as a component of Robert Montgomery’s thesis work.
