# Supplementary material for: Constrained optimization: evaluating possible packages of community health interventions with competing resource requirements in Galmudug, Somalia
Source: Health Policy Plan. 2025 Mar 11;40(5):566–77. doi: 10.1093/heapol/czaf014 (PMC12063593; doi:10.1093/heapol/czaf014)
Supplement: czaf014_Supp [file czaf014_supp.zip › OnlineAppendix.pdf]

Appendices

A Constrained Optimization in Health Settings

In the nonprofit and global health spaces, there has been a concerted effort to measure cost-weighted impact in resource-constrained environments. Economics and Operations expertise have been a welcome addition to this effort, and there are partnerships, of which this paper is one, to expand tools, such as constrained optimization, into decision-making within this space. Examples of articles either providing or advocating for the incorporation of similar tools to the one we design include Marseille *et al.* (2015), Ochalek *et al.* (2019), and Stuart *et al.* (2023).

As health program designers place more emphasis on country-specific approaches, reviews of existing programs have often shown that intervention selection is largely arbitrary. In a review of neonatal programs and surveys, Haws et al. (2007) find that interventions are "largely bundled out of convenience or funding requirements, rather than based on anticipated synergistic effects." Similar attempts to formalize the trade-offs between different interventions can be found in Nandi *et al.* (2016), which measures the increase in lives saved when scaling at-home neonatal care packages. Likewise, Darmstadt *et al.* (2005) constructs a general neonatal package of care with cost-efficacy analysis in order to show the effectiveness of at-home neonatal treatments. Additionally, Stinnett and Paltiel (1996), Ochalek *et al.* (2018), van Baal *et al.* (2018), and Karsu and Morton (2020) are all examples of works that apply optimization techniques to the distribution of limited healthcare resources.

B Optimization Technique

We now discuss the methods used to solve the optimization model. As noted earlier, the model is formulated as a mixed integer non-linear program. To solve the model, we rely on the techniques used by Qin *et al.* (2022). We provide an abbreviated version of their process,

with slight adjustments made to match our problem; however, we recommend the reader refer to their paper for a full accounting of the process. In their paper, they require a fast method for solving an integer program for automatic guided vehicles transporting shelves within a warehouse. To do so, they show that their problem is separable when a Lagrangian relaxation is introduced. As their program and ours share a similar structure, we can follow the same steps to create two subproblems (one for each set of decision variables x and y) and solve them in sequential order.

For convenience, we define the *mixed-integer program for maternal and newborn healthcare optimization* comprised of Equations (1)-(7) as (P), and $V(P)$ as the optimal objective value of the problem (P). We introduce $\lambda := \{\lambda_j \geq 0, j \in J\}$ as the Lagrangian multipliers associated with constraint (5). This allows us to write the following partially relaxed problem:

$\hat{P}(\Lambda)$:

$$\max_{x,y}\ D_n \sum_{i=1}^{8} r_i \Big[1 - \prod_{j=1}^{J} \big(1 - f_{ij} \frac{e_{ij} x_j}{1 - e_{ij} c_j}\big)\Big] + D_M \sum_{i=9}^{17} r_i \Big[1 - \prod_{j=1}^{J} (1 - f_{ij} \frac{e_{ij} x_j}{1 - e_{ij} c_j})\Big] - \sum_{j=1}^{J} \lambda_j \big(x_j - y_j\big)$$

subject to

$$Ax \leq a \tag{12}$$

$$By \leq b \tag{13}$$

$$x_j \leq \min(u_j - c_j,\ d,\ \beta_j/\alpha_j),\ \forall j \tag{14}$$

$$y_j \in \{0,1\},\ \forall\ j \tag{15}$$

$$x_j \geq 0,\ \forall\ j \tag{16}$$

Under duality, for any Λ we have that $V(\hat{P}(\Lambda))$ is an upper bound on the original problem $V(P)$, meaning $V(\hat{P}(\Lambda)) \geq V(P)$. The optimal dual variable Λ^*, is given by $\Lambda^* = \arg\min_{\Lambda} V(\hat{P}(\Lambda))$.

For any Λ, x_j and y_j become totally separable within the problem $(\hat{P}(\Lambda))$. Because of this separability, any solution to the two subproblems $(P^1(\Lambda))$ and $(P^2(\Lambda))$, is also a solution to $(\hat{P}(\Lambda))$. However, this solution may violate constraint Equation (5) of problem

(P), and thus may be infeasible for the original problem of interest.

$$P^1(\Lambda):\ \max_x\ D_n \sum_{i=1}^{8} r_i\big[1 - \prod_{j=1}^{J}\big(1 - f_{ij}\frac{e_{ij}x_j}{1-e_{ij}c_j}\big)\big] + D_M \sum_{i=9}^{17} r_i\big[1 - \prod_{j=1}^{J}(1 - f_{ij}\frac{e_{ij}x_j}{1-e_{ij}c_j})\big] - \sum_{j=1}^{J}\lambda_j x_j$$

subject to

$$Ax \leq a \tag{17}$$

$$x_j \leq \min(u_j - c_j,\ d,\ \beta_j/\alpha_j),\ \forall j \tag{18}$$

$$x_j \geq 0,\ \forall\ j \tag{19}$$

$$P^2(\Lambda):\ \max_y\ \sum_{j=1}^{J}\lambda_j y_j$$

subject to

$$By \leq b \tag{20}$$

$$y_j \in \{0,1\},\ \forall\ j \tag{21}$$

To arrive at a feasible solution to (P), we approach this problem by solving for problem (P^2) and using the solution for y^* to solve (P^1), which is a modified version of $(P^1(\Lambda))$, and is written as follows:

$$P^1\ \max_x\ D_n \sum_{i=1}^{8} r_i\big[1 - \prod_{j=1}^{J}\big(1 - f_{ij}\frac{e_{ij}x_j}{1-e_{ij}c_j}\big)\big] + D_M \sum_{i=9}^{17} r_i\big[1 - \prod_{j=1}^{J}(1 - f_{ij}\frac{e_{ij}x_j}{1-e_{ij}c_j})\big]$$

subject to

$$Ax \leq a \tag{22}$$

$$x_j \leq \min(u_j - c_j,\ d,\ \beta_j/\alpha_j),\ \forall j \tag{23}$$

$$x_j \geq 0,\ \forall\ j \tag{24}$$

$$x_j \leq y_j^*\ \forall j \tag{25}$$

C The Lives Saved Tool

One of the challenges with modeling healthcare operations in developing countries is the lack of reliable, context-specific data. We address this issue by incorporating data from the Lives Saved Tool (LiST), a combined effort by the Johns Hopkins University Bloomberg School of Public Health and the Bill & Melinda Gates Foundation. This tool is widely used by global health researchers, with over 100 peer-reviewed studies that use LiST, and several studies that test the estimates generated by LiST with measured mortality reduction in implemented settings (Walker *et al.* 2013). LiST provides both equations to predict the number of deaths averted from increases in coverage for various health interventions, and a set of demographic and health data to populate these equations in different settings. Reliance on LiST sets a clear objective of maximizing lives saved in favor of other health metrics such as reducing DALYs and QALYs. We find this to be an appropriate objective for our work; however, we do acknowledge an ongoing debate about the strengths and shortcomings of this target, see Spigelman *et al.* (2018).

The Lives Saved Tool (LiST) was developed as an evaluation aid for health program design. It is widely used in the global health community, has buy-in from several country governments with community health programs, is transparent in its methods and calculations, and is regularly updated. In this section, we outline the data within LiST that are used as parameters for the model. These groups of parameters are listed in Table 9, with further descriptions provided below. The reader is referred to Walker *et al.* (2013) for further details of the methodology used in LiST.

Table 9: Country specific parameters from LiST used in the model.

1. The current level of coverage for each intervention
2. The annual number of deaths in each population group
3. The percentage of deaths attributed to each cause of death for each population group
4. Efficacy of each intervention for each cause of death
5. Affected fraction of each intervention for each cause of death

LiST provides an estimate for the current level of coverage for each intervention, displayed as a percentage of the country's population. By subtracting the current level of coverage from the maximum percentage of the population that could reasonably be expected to receive an intervention, referred to as full coverage and often listed as 90%, we arrive at the percentage of the population available to receive that intervention. This difference between full coverage and current coverage provides a cap on the available increase in coverage. In doing so, it ensures that the number of possible lives saved for each intervention matches the number of deaths, for which the intervention addresses the underlying cause, within the untreated portion of the population.

LiST separates outcomes into four population groups: Maternal, Neonatal (0 to 1 month), Stillbirth, and Children (1 to 59 months). It uses a mortality rate for each population group, along with the projected size of each group, to estimate the number of deaths within each group. These deaths within each group are further categorized by cause of death, for which LiST provides country-specific percentages based on demographic and public health surveys.

LiST identifies nine maternal causes of death and eight neonatal causes of death. These are shown for the Somalia setting in Tables 11 and 12 of Appendix D. Each intervention then addresses at least one, and in many cases several, of these causes of death. Although the model we provide is intended for use in the design and evaluation of maternal and neonatal programs, the availability of country-specific mortality rates for children and stillbirths allows for a straightforward extension of this model to health programs focusing on these additional populations.

To calculate the number of lives saved attributed to each intervention, LiST provides affected fraction and efficacy values for each intervention and cause-of-mortality pair. The affected fraction of an intervention is defined in LiST as the proportion of cause-specific deaths that are susceptible to treatment with a given intervention (Walker *et al.* 2013). Likewise, the efficacy of an intervention is the proportion of cause-specific deaths that can be averted with that intervention. In other words, the affected fraction measures what

proportion of a cause of death a specific intervention targets, and efficacy measures how well an intervention works by reducing the relevant fraction for that cause of death.

D Somalia Model Parameters

We proceed by defining the list of model parameters used in the Somalia setting. The target populations for Somalia are shown in Table 10 and the mortality rates by cause are shown in Tables 11 and 12. The matrices of neonatal affected fractions and efficacy values for each intervention and cause of death pair are shown in Tables 13 and 14. We then provide matrices for maternal affected fractions and efficacy values in Tables 15 and 16. The values in these matrices match those provided by LiST, except for the italicized values, which were adjusted to reflect the input from Somali health professionals. In Tables 15 and 16 we leave out the columns for antepartum hemorrhage and intrapartum hemorrhage, as, in LiST's calculations, none of the interventions considered impact those causes of death.

Table 10: Target populations in Somalia

Target population	Size
Pregnant women	973,781
Live births	716,518
Number of households	2,935,990
Pregnancies carried to term	843,109
Children 0-59 months	3,036,352
Abortions	130,672

Table 11: Somalia neonatal mortality by cause

Cause of death	Percent of neonatal deaths by cause	2023 projected deaths by cause
Diarrhea	0.94%	248
Sepsis	9.88%	2,609
Pneumonia	9.58%	2,530
Asphyxia	37.05%	9,785
Prematurity	22.62%	5,974
Tetanus	6.41%	1,693
Congenital anomalies	6.61%	1,745
Other	6.92%	1,827

Table 12: Somalia maternal mortality by cause

Cause of death	Percent of maternal deaths by cause	2023 projected deaths by cause
Antepartum hemorrhage	8.95%	443
Intrapartum hemorrhage	0.88%	43
Postpartum hemorrhage	15.71%	778
Hypertensive disorders	16.94%	840
Sepsis	10.54%	522
Abortion	10.97%	543
Embolism	1.89%	93
Other direct causes	8.46%	419
Indirect causes	25.66%	1,272

Table 13: Neonatal affected fraction

Intervention	Diarrhea	Sepsis	Pneumonia	Asphyxia	Prematurity	Tetanus	Congenital anomalies	Other
Tetanus toxoid vaccination	0	0	0	0	0	1	0	0
Multiple micronutrient supplementation	0	0.5	0.5	0.5	0.5	0	0	0
Iron supplementation in pregnancy	0	0	0	0	0	0	*0.200*	0
Calcium supplementation	0	0	0	0	1	0	0	0
Balanced energy supplementation	0	0.5	0.5	0.5	0.5	0	0	0
KMC - Kangaroo mother care	0	0	0	0	*0.580*	0	0	0
Breastfeeding promotion	0.5	0.5	0.5	0	0	0	0	0
Hand washing with soap	1	0	0	0	0	0	0	0
Prevention of malaria in pregnancy	0	0.5	0.5	0.5	0.5	0	0	0
Basic sanitation	1	0	0	0	0	0	0	0
Clean birth environment	0	1	0	0	0	1	0	0
Clean cord care	0	1	0	0	0	1	0	0
Immediate drying and additional stimulation	0	0	0	1	1	0	0	0
Neonatal resuscitation	0	0	0	1	1	0	0	0
Uterotonics for postpartum hemorrhage	0	0	0	0	0	0	0	0
Syphilis detection and treatment	0	0.5	0.5	0.5	0.5	0	0	0
Thermal regulation	0	1	0	0	1	0	0	0
Manual removal of placenta	0	0	0	0	0	0	0	0
Point-of-use filtered water	1	0	0	0	0	0	0	0
Malaria case management	0	0	0	0	0	0	0	0
Injectable antibiotics for neonatal sepsis	0	1	1	0	0	0	0	0
Safe abortion services	0	0	0	0	0	0	0	0
Oral antibiotics for neonatal sepsis	0	1	1	0	0	0	0	0
Post abortion case management	0	0	0	0	0	0	0	0
Antibiotics for treatment of dysentery	0.120	0	0	0	0	0	0	0

Table 14: Neonatal effectiveness

Intervention	Diarrhea	Sepsis	Pneumonia	Asphyxia	Prematurity	Tetanus	Congenital anomalies	Other
Tetanus toxoid vaccination	0	0	0	0	0	0.940	0	0
Multiple micronutrient supplementation	0	0.058	0.059	0.058	0.100	0	0	0
Iron supplementation in pregnancy	0	0	0	0	0	0	0.460	0
Calcium supplementation	0	0	0	0	0.120	0	0	0
Balanced energy supplementation	0	0.097	0.096	0.096	0.074	0	0	0
KMC - Kangaroo mother care	0	0	0	0	0.510	0	0	0
Breastfeeding promotion	0.040	0.041	0.041	0	0	0	0	0
Hand washing with soap	0.070	0	0	0	0	0	0	0
Prevention of malaria in pregnancy	0	0.063	0.063	0.063	0.148	0	0	0
Basic sanitation	0.160	0	0	0	0	0	0	0
Clean birth environment	0	0.270	0	0	0	0.380	0	0
Clean cord care	0	0.400	0	0	0	0.400	0	0
Immediate drying and additional stimulation	0	0	0	0.100	0.100	0	0	0
Neonatal resuscitation	0	0	0	0.300	0.100	0	0	0
Uterotonics for postpartum hemorrhage	0	0	0	0	0	0	0	0
Syphilis detection and treatment	0	0.011	0.010	0.010	0.023	0	0	0
Thermal regulation	0	0.100	0	0	0.200	0	0	0
Manual removal of placenta	0	0	0	0	0	0	0	0
Point-of-use filtered water	0.400	0	0	0	0	0	0	0
Malaria case management	0	0	0	0	0	0	0	0
Injectable antibiotics for neonatal sepsis	0	0.650	0.750	0	0	0	0	0
Safe abortion services	0	0	0	0	0	0	0	0
Oral antibiotics for neonatal sepsis	0	0.299	0.452	0	0	0	0	0
Post abortion case management	0	0	0	0	0	0	0	0
Antibiotics for treatment of dysentery	0.820	0	0	0	0	0	0	0

Table 15: Maternal affected fraction

Intrapartum hemorrhage	Postpartum hemorrhage	Hypertensive disorders	Sepsis	Abortion	Embolism	Other direct causes	Indirect causes
Tetanus toxoid vaccination	0	0	0	0	0	0	0.005
Multiple micronutrient supplementation	0	0	0	0	0	0	0
Iron supplementation in pregnancy	0	0	0	0	0	0	0
Calcium supplementation	0	1	0	0	0	0	0
Balanced energy supplementation	0	0	0	0	0	0	0
KMC - Kangaroo mother care	0	0	0	0	0	0	0
Breastfeeding promotion	0	0	0	0	0	0	0
Hand washing with soap	0	0	0	0	0	0	0
Prevention of malaria in pregnancy	0	0	0	0	0	0	0.045
Basic sanitation	0	0	0	0	0	0	0
Clean birth environment	0	0	1	0	0	0	0
Clean cord care	0	0	0	0	0	0	0
Immediate drying and additional stimulation	0	0	0	0	0	0	0
Neonatal resuscitation	0	0	0	0	0	0	0
Uterotonics for postpartum hemorrhage	1	0	0	0	0	0	0
Syphilis detection and treatment	0	0	0	0	0	0	0
Thermal regulation	0	0	0	0	0	0	0
Manual removal of placenta	1	0	0	0	0	0	0
Point-of-use filtered water	0	0	0	0	0	0	0
Malaria case management	0	0	0	0	0	0	0.045
Injectable antibiotics for neonatal sepsis	0	0	0	0	0	0	0
Safe abortion services	0	0	0	0.816	0	0	0
Oral antibiotics for neonatal sepsis	0	0	0	0	0	0	0
Post abortion case management	0	0	0	0.816	0	0	0
Antibiotics for treatment of dysentery	0	0	0	0	0	0	0

Table 16: Maternal effectiveness

Intervention	Postpartum hemorrhage	Hypertensive disorders	Sepsis	Abortion	Embolism	Other direct causes	Indirect causes
Tetanus toxoid vaccination	0	0	0	0	0	0	0.980
Multiple micronutrient supplementation	0	0	0	0	0	0	0
Iron supplementation in pregnancy	0	0	0	0	0	0	0
Calcium supplementation	0	0.200	0	0	0	0	0
Balanced energy supplementation	0	0	0	0	0	0	0
KMC - Kangaroo mother care	0	0	0	0	0	0	0
Breastfeeding promotion	0	0	0	0	0	0	0
Hand washing with soap	0	0	0	0	0	0	0
Prevention of malaria in pregnancy	0	0	0	0	0	0	0.725
Basic sanitation	0	0	0	0	0	0	0
Clean birth environment	0	0	0.600	0	0	0	0
Clean cord care	0	0	0	0	0	0	0
Immediate drying and additional stimulation	0	0	0	0	0	0	0
Neonatal resuscitation	0	0	0	0	0	0	0
Uterotonics for postpartum hemorrhage	0.775	0	0	0	0	0	0
Syphilis detection and treatment	0	0	0	0	0	0	0
Thermal regulation	0	0	0	0	0	0	0
Manual removal of placenta	0.300	0	0	0	0	0	0
Point-of-use filtered water	0	0	0	0	0	0	0
Malaria case management	0	0	0	0	0	0	0.800
Injectable antibiotics for neonatal sepsis	0	0	0	0	0	0	0
Safe abortion services	0	0	0	0.950	0	0	0
Oral antibiotics for neonatal sepsis	0	0	0	0	0	0	0
Post abortion case management	0	0	0	0.800	0	0	0
Antibiotics for treatment of dysentery	0	0	0	0	0	0	0

In Table 17, we display the maximum achievable coverage for each intervention with a fixed number of 1,450 FHWs. The calculation for these values is described in the Methods Section.

Table 17: Maternal and neonatal interventions

Intervention	Max allowable coverage with 1,450 FHWs (%)
Tetanus toxoid vaccination	76.88
Multiple micronutrient supplementation	9.88
Iron supplementation in pregnancy	9.88
Calcium supplementation	9.88
Balanced energy supplementation	9.88
KMC - Kangaroo mother care	15.23
Breastfeeding promotion	18.69
Hand washing with soap	19.71
Prevention of malaria in pregnancy	12.36
Basic sanitation	48.21
Clean birth environment	16.55
Clean cord care	18.15
Immediate drying and additional stimulation	17.61
Neonatal resuscitation	13.61
Uterotonics for postpartum hemorrhage	17.24
Syphilis detection and treatment	13.65
Thermal regulation	19.06
Manual removal of placenta	12.38
Point-of-use filtered water	14.37
Malaria case management	11.16
Injectable antibiotics for neonatal sepsis	19.24
Safe abortion services	13.18
Oral antibiotics for neonatal sepsis	9.88
Post abortion case management	9.88
Antibiotics for treatment of dysentery	9.88

As outlined in the Methods Section, we need to provide estimates for the number of treatments required to reach full coverage. To do so, in Table 18, for each intervention, we provide the title of the target population, the size of the target population, the percentage of the target population requiring treatment, and the resulting number of treatments needed to reach full coverage. As before, the values in Table 18 are provided by LiST, except for the italicized values, which were once again suggested by health professionals in Somalia.

Table 18: Calculations for the estimated number of treatments

Intervention	Target population	Size	Requiring treatment (%)	Treatments needed
Tetanus toxoid vaccination	Pregnant women	973,781	100	973,781
Multiple micronutrient supplementation	Pregnant women	973,781	100	973,781
Iron supplementation in pregnancy	Pregnant women	973,781	100	973,781
Calcium supplementation	Pregnant women	973,781	100	973,781
Balanced energy supplementation	Pregnant women	973,781	7	65,243
KMC - Kangaroo mother care	Live births	716,518	12	85,982
Breastfeeding promotion	Live births	716,518	100	716,518
Hand washing with soap	Number of households	3,320,000	100	3,320,000
Prevention of malaria in pregnancy	Pregnant women	973,781	100	973,781
Basic sanitation	Number of households	3,320,000	100	3,320,000
Clean birth environment	Pregnancies carried to term	843,109	100	843,109
Clean cord care	Live births	716,518	100	716,518
Immediate drying and additional stimulation	Live births	716,518	100	716,518
Neonatal resuscitation	Live births	716,518	7	50,156
Uterotonics for postpartum hemorrhage	Pregnancies carried to term	843,109	1	10,117
Syphilis detection and treatment	Pregnant women	973,781	100	973,781
Thermal regulation	Live births	716,518	100	716,518
Manual removal of placenta	Pregnancies carried to term	843,109	2	16,862
Point-of-use filtered water	Number of households	3,320,000	100	3,320,000
Malaria case management	Pregnant women	973,781	20	194,756
Injectable antibiotics for neonatal sepsis	Live births	716,518	8	54,455
Safe abortion services	Abortions	130,672	100	130,672
Oral antibiotics for neonatal sepsis	Live births	716,518	8	54,455
Post abortion case management	Abortions	130,672	24	31,623
Antibiotics for treatment of dysentery	Children 0-59 months	101,211	10	10,121

E Expanded Versions of the Model

We now provide the results for the expanded model discussed in the Results Section. Table 19 includes the results of the model permitting 22 of the 25 community-level interventions, with the two interventions related to abortion services and the manual removal of placenta intervention not considered. The objective function of this solution has a value of 1,990 projected lives saved or 48% of maternal and neonatal deaths in Galmudug state in 2023. The amounts of each constraint resource used in the model solution are listed in Table 20.

Table 19: Optimal package of interventions with relaxed restrictions for the Somalia setting

Intervention	y_j	Coverage Change (x_j)	Coverage Target	Current Coverage
Tetanus toxoid vaccination	1	9.88%	76.88%	67%
MM supplementation in pregnancy	1	9.88%	9.88%	0%
Iron supplementation in pregnancy	0		0%	0%
Calcium supplementation	0		0%	0%
Balanced energy supplementation	1	9.88%	9.88%	0%
KMC - Kangaroo mother care	1	9.88%	14.88%	5%
Breastfeeding promotion	0		9%	9%
Hand washing with soap	0		10%	10%
Prevention of malaria in pregnancy	1	9.80%	11.80%	2%
Basic sanitation	1	9.88%	47.88%	38%
Clean birth environment	1	9.88%	16.88%	7%
Clean cord care	1	9.88%	17.88%	8%
Immediate drying and stimulation	1	9.88%	17.88%	8%
Neonatal resuscitation	0		4%	4%
Uterotonics for postpartum hemorrhage	0		7%	7%
Syphilis detection and treatment	0		4%	4%
Thermal regulation	1	9.88%	18.88%	9%
Manual removal of placenta	0		3%	3%
Point-of-use filtered water	0		4%	4%
Malaria case management	0		1%	1%
Injectable antibiotics for neonatal sepsis	1	9.88%	18.88%	9%
Safe abortion services	0		3%	3%
Oral antibiotics for neonatal sepsis	1	9.88%	9.88%	0%
Post abortion case management	0		0%	0%
Antibiotics for treatment of dysentery	0		0%	0%

Table 20: Constraint budget usage for expanded Somalia program

Constraint	Budget	Usage
Total time available per month for care	22,602 hours	16,828 hours
Total supervisor time per month for training	3,927 hours	1,184 hours
Total commodity budget available for scaling interventions	$ 435,000	$ 435,000
Total time available in curriculum	1,200 mins	1,170 mins

We now consider a version of the model where we perform the same analysis we did in Table 19, except with interventions involving injections removed. As such, tetanus toxoid vaccination, syphilis detection and treatment, manual removal of placenta, injectable antibi-

otics for neonatal sepsis, safe abortion services, and post-abortion case management are not eligible for inclusion. The results are shown in Table 21. The objective function for the optimal solution has a value of $1,335$ lives saved.

Table 21: Optimal package of interventions with relaxed restrictions excluding injections

Intervention	y_j	x_j: Coverage change (%)	Coverage target (%)	Current coverage (%)
Tetanus toxoid vaccination	0		67	67
MM supplementation in pregnancy	1	9.88	9.88	0
Iron supplementation in pregnancy	0		0	0
Calcium supplementation	0		0	0
Balanced energy supplementation	1	9.88	9.88	0
KMC - Kangaroo mother care	1	9.88	14.88	5
Breastfeeding promotion	0		9	9
Hand washing with soap	0		10	10
Prevention of malaria in pregnancy	1	9.88	11.88	2
Basic sanitation	0		38	38
Clean birth environment	1	9.88	16.88	7
Clean cord care	1	9.88	17.88	8
Immediate drying and stimulation	1	9.88	17.88	8
Neonatal resuscitation	0		4	4
Uterotonics for postpartum hemorrhage	1	9.88	16.88	7
Syphilis detection and treatment	0		4	4
Thermal regulation	1	9.88	18.88	9
Manual removal of placenta	0		3	3
Point-of-use filtered water	1	0.13	4.13	4
Malaria case management	0		1	1
Injectable antibiotics for neonatal sepsis	0		9	9
Safe abortion services	0		3	3
Oral antibiotics for neonatal sepsis	1	9.88	9.88	0
Post abortion case management	0		0	0
Antibiotics for treatment of dysentery	0		0	0

F Expanded Sensitivity Analysis

As intervention efficacy plays an important role in the model's objective function, it is worth measuring the extent to which the results of this model are driven by assumptions about the efficacy of community-level delivery. The LiST definitions for each intervention can be quite specific, and in some cases where the interventions are adapted to local contexts, they may be done so in a way that does not adhere to the exact definitions' specifications. The data provided by LiST is mostly informed by studies of efficacy in health facility-type settings.

Many of these interventions can reasonably be expected to perform as well when delivered in the field; however, there are several for which the procedures would need to be altered to be provided in a community setting.

As a means to better understand how differences between efficacy in the field compared to efficacy at the health facility could alter the projected number of lives saved and the composition of the optimal solution of the model, we conduct sensitivity analysis on the six interventions we feel could differ in their efficacy between the two settings. These interventions are kangaroo mother care, clean cord care, immediate drying and additional stimulation, neonatal resuscitation, uterotonics for postpartum hemorrhage, and thermal regulation.

In Table 22, we list the interventions identified for this analysis and their efficacy values for the health facility setting. We then indicate whether or not an intervention is included in the optimal package of interventions shown in Table 19. Using the same constraints and the expanded list of permissible interventions, we re-run the model for each intervention with the efficacy values for that intervention adjusted down by 50% then by 75%, holding the efficacy values of the other interventions in the model unchanged. We then indicate if the intervention is still included in the optimal set of interventions, and how many lives saved the model predicts will result from this change.

Table 22: Intervention efficacy sensitivity analysis

Intervention	Current efficacy	Included with current efficacy	0.5×Efficacy included	Lives saved	0.25×Efficacy included	Lives saved
Kangaroo mother care	0.510	Yes	Yes	1,903	No	1,878
Clean cord care	0.400*	Yes	Yes	1,918	Yes	1,883
Immediate drying and additional stimulation	0.100*	Yes	Yes	1,914	Yes	1,902
Neonatal resuscitation	0.300*	No	No	1,990	No	1,990
Uterotonics for postpartum hemorrhage	0.775	No	No	1,990	No	1,990
Thermal regulation	0.200*	Yes	Yes	1,922	No	1,914

We place an asterisk next to the efficacy values of the interventions that impact multiple causes of death. For these interventions, we list the larger of the two efficacy values (all of these interventions affect at most two causes of death). However, when we perform the sensitivity analysis for each intervention we adjust all the efficacy values for that intervention

by the same weight. For example, thermal regulation has an efficacy value of 0.1 on the neonatal sepsis cause of death and an efficacy value of 0.2 on the neonatal prematurity cause of death. When we run the model with 50% of the stated efficacy value of thermal regulation, we adjust both thermal regulation's efficacy value on neonatal sepsis to 0.05 and its efficacy value on neonatal prematurity to 0.1 at the same time.

Of the six interventions identified, four are included in the optimal set of interventions from Table 19. We do not perform sensitivity analysis on the two interventions not included (neonatal resuscitation and uterotonics for postpartum hemorrhage), as they will still not be included in the set of optimal interventions once their efficacy values are reduced. With a baseline of 1,990 lives saved, we see that all four of the interventions included in the original optimal set are still included when their efficacy values are reduced by 50%. Once the efficacy of these interventions is reduced by 75%, we see that kangaroo mother care and thermal regulation are removed from the set of optimal interventions, whereas clean cord care and immediate drying and additional stimulation remain in the optimal set. This analysis should prove useful to practitioners who express uncertainty about the relative efficacy of these interventions when performed in the field as opposed to in a health facility setting. Furthermore, this analysis can be extended to other interventions and allows for finer gradations of efficacy adjustments.

G Somalia Model Constraints

To encourage the application of the model to additional settings, we provide detailed calculations for the constraints used in the Somalia setting. We begin with the time required by FHWs to deliver interventions. In order to calculate the amount of time each intervention requires, we multiply the number of treatments needed to reach full coverage for that intervention by the frequency of providing that intervention and the amount of time each treatment takes to provide. We then divide this number by twelve to produce the time per month required to reach full coverage of an intervention. The Somalia setting values for this

calculation are provided in Table 23.

The first constraint in our model is the total time available to offer maternal and neonatal services per month. As discussed earlier, the overtasking of FHWs often makes available time one of the limiting factors to providing adequate care. There is a certain degree of flexibility in how the FHWs divide their time between households. It is reasonable to expect that FHWs will prioritize households with neonates, pregnant women, and new mothers, while still attending to their required number of households per month. It would thus seem improper to place a strict time constraint on MNH care. However, if one were to show a list of interventions to an FHW that she is expected to provide as part of her service package, she would likely be able to say right away whether the number of interventions is reasonable, too many, or too few. We attempt to capture this, to a degree, in the time constraint. In contrast to the budget for funding, the interpretation of the time constraint should be less rigid. The uncertainty around this constraint emphasizes the need to have buy-in from all levels of the program design. The community providers and FHWs will have a better feel for what is reasonable for their setting and thus need to be part of an iterative modeling process, whereby feedback informs changes to the implementation of the model's insights if not the model overall.

The right-hand side of the constraint (or time budget) is calculated by multiplying together the number of FHWs (1,450), by the time allotted for MNH care which is given by the time FHWs spend on tasks per month (6 days x 4 hours x 4.33 weeks) minus the travel time per month (60 mins per day) and time spent on non-MNH tasks. We allow for the time spent on non-MNH tasks to be represented as the percentage of time spent on non-MNH responsibilities (60%). We lay out the calculation explicitly so that adjustments to individual parameters can be easily made to match a given setting.

$$1450\big[(6 \times 240 \times 4.33) - \big(6 \times 60 \times 4.33 + 0.6\big(6 \times 240 \times 4.33\big)\big)\big] = 1{,}356{,}156 \text{ minutes/month} \tag{26}$$

1,356,156 minutes per month is equivalent to 22,602 hours per month, or 15.59 hours per

FHW per month for MNH care.

Table 23: Time requirements for providing interventions

Intervention	Number of treatments needed	Annual frequency	Time to provide intervention (mins)	Monthly full coverage requirement (mins)
Tetanus toxoid vaccination	973,781	2	5	811,484
Multiple micronutrient supplementation	973,781	1	10	811,484
Iron supplementation in pregnancy	973,781	1	8	649,187
Calcium supplementation	973,781	1	8	649,187
Balanced energy supplementation	65,243	1	8	43,496
KMC - Kangaroo mother care	85,982	1	40	286,607
Breastfeeding promotion	716,518	1	30	1,791,295
Hand washing with soap	3,320,000	1	5	1,383,333
Prevention of malaria in pregnancy	973,781	1	5	405,742
Basic sanitation	3,320,000	1	5	1,383,333
Clean birth environment	843,109	1	60	4,215,545
Clean cord care	716,518	1	15	895,648
Immediate drying and additional stimulation	716,518	1	10	597,098
Neonatal resuscitation	50,156	1	20	83,594
Uterotonics for postpartum hemorrhage	10,117	1	90	75,880
Syphilis detection and treatment	973,781	1	15	1,217,226
Thermal regulation	716,518	1	30	1,791,295
Manual removal of placenta	16,862	1	100	140,518
Point-of-use filtered water	3,320,000	1	10	2,766,667
Malaria case management	194,756	1	15	243,445
Injectable antibiotics for neonatal sepsis	54,455	1	40	181,518
Safe abortion services	130,672	1	30	326,680
Oral antibiotics for neonatal sepsis	54,455	1	5	22,690
Post abortion case management	31,623	1	90	237,170
Antibiotics for treatment of dysentery	10,121	1	2	1,687

We next calculate the supervision time available to oversee FHW in-service training. To calculate the amount of supervision time each intervention requires, we multiply the number of observations for each intervention by the time needed per observation, and the number of FHWs (1,450). These calculations are shown in Table 24.

Table 24: Supervision time required to certify intervention proficiency

Intervention	Observations required (per year)	Time per observation (mins)	Time required for full coverage (mins)
Tetanus toxoid vaccination	12	5	87,000
Multiple micronutrient supplementation	24	10	348,000
Iron supplementation in pregnancy	24	8	278,400
Calcium supplementation	24	8	278,400
Balanced energy supplementation	24	8	278,400
KMC - Kangaroo mother care	24	50	1,740,000
Breastfeeding promotion	12	40	696,000
Hand washing with soap	12	5	87,000
Prevention of malaria in pregnancy	12	5	87,000
Basic sanitation	12	5	87,000
Clean birth environment	12	60	1,044,000
Clean cord care	24	30	1,044,000
Immediate drying and additional stimulation	12	30	522,000
Neonatal resuscitation	12	60	1,044,000
Uterotonics for postpartum hemorrhage	24	90	3,132,000
Syphilis detection and treatment	24	20	696,000
Thermal regulation	24	50	1,740,000
Manual removal of placenta	24	100	3,480,000
Point-of-use filtered water	12	15	261,000
Malaria case management	48	15	1,044,000
Injectable antibiotics for neonatal sepsis	24	40	1,392,000
Safe abortion services	24	30	1,044,000
Oral antibiotics for neonatal sepsis	24	5	174,000
Post abortion case management	48	30	2,088,000
Antibiotics for treatment of dysentery	24	2	69,600

The right-hand side of the constraint (or supervision time budget) is calculated by multiplying the number of supervisors by the minutes per year each supervisor has to observe or support FHWs. We assume that each supervisor has 4 hours a day for supervision tasks, works 5 days a week, and spends 25% of her time traveling and 50% of the remaining time on MNH supervision. In Somalia, there is a supervisor for every 12 CHWs. The calculation of the supervision time budget is shown in Equation (27).

$$\frac{1450}{12} \times 0.5(1 - 0.25)(5 \times 240 \times 52) = 2{,}827{,}500 \text{ minutes per year} \tag{27}$$

2,827,500 minutes per year is equivalent to 3,927 hours per month, or 32.50 hours per supervisor per month for MNH care.

We now calculate the costs of increasing the coverage for each intervention. To calculate the cost to offer each intervention at full coverage, we multiply the number of treatments needed for full coverage by the cost of one unit of that treatment's required commodities and the number of commodities needed per treatment. These calculations for the Somalia setting are shown in Table 25. The annual budget to scale interventions ($435,000), which serves as the right-hand side of the constraint, is provided as part of the pilot program.

Table 25: Costs to increase coverage of interventions

Intervention	Treatments	Cost per commodity ($)	Commodities per treatment	Total cost ($)
Tetanus toxoid vaccination	973,781	0.22	2	428,464
Multiple micronutrient supplementation	973,781	0.50	1	486,891
Iron supplementation in pregnancy	973,781	0.04	150	5,842,686
Calcium supplementation	973,781	11.00	1	10,711,591
Balanced energy supplementation	65,243	5.00	1	326,217
KMC - Kangaroo mother care	85,982	0	1	0
Breastfeeding promotion	716,518	0	1	0
Hand washing with soap	3,320,000	0	1	0
Prevention of malaria in pregnancy	973,781	2.00	1	1,947,562
Basic sanitation	3,320,000	0	1	0
Clean birth environment	843,109	1.37	1	1,155,059
Clean cord care	716,518	0	1	0
Immediate drying and additional stimulation	716,518	0	1	0
Neonatal resuscitation	50,156	0.38	1	19,059
Uterotonics for postpartum hemorrhage	10,117	0.23	1	2,327
Syphilis detection and treatment	973,781	4.39	1	4,274,899
Thermal regulation	716,518	0	1	0
Manual removal of placenta	16,862	3.99	1	67,280
Point-of-use filtered water	3,320,000	10.00	1	33,200,000
Malaria case management	194,756	2.55	1	496,628
Injectable antibiotics for neonatal sepsis	54,455	1.24	1	67,525
Safe abortion services	130,672	1.26	1	164,647
Oral antibiotics for neonatal sepsis	54,455	0.12	1	6,535
Post abortion case management	31,623	15.60	1	493,313
Antibiotics for treatment of dysentery	10,121	0.12	1	1,215

Now that we have covered the three constraints that comprise Matrix A and Vector a of Equation (2), we move to Equation (3) and provide the values for the Somalia setting in Matrix B and Vector b. The first constraint in Matrix B includes the curriculum time required for each intervention. We list these values in Table 26. The amount of curriculum time available to teach MNH care (1,200 minutes) is provided by the Ministry of Health.

Table 26: Curriculum time required to teach maternal and neonatal interventions

Intervention	Curriculum time (minutes)
Tetanus toxoid vaccination	60
Multiple micronutrient supplementation	60
Iron supplementation in pregnancy	60
Calcium supplementation	60
Balanced energy supplementation	60
KMC - Kangaroo mother care	120
Breastfeeding promotion	180
Hand washing with soap	120
Prevention of malaria in pregnancy	60
Basic sanitation	60
Clean birth environment	180
Clean cord care	60
Immediate drying and additional stimulation	240
Neonatal resuscitation	960
Uterotonics for postpartum hemorrhage	180
Syphilis detection and treatment	120
Thermal regulation	120
Manual removal of placenta	240
Point-of-use filtered water	10
Malaria case management	45
Injectable antibiotics for neonatal sepsis	120
Safe abortion services	240
Oral antibiotics for neonatal sepsis	60
Post abortion case management	240
Antibiotics for treatment of dysentery	60

The application of the model to Somalia does not include considerations for a budget to launch interventions and is thus not included in this Appendix. Additionally, the government permission status for each intervention is included in Table 3 of the Methods Section.

H Ethiopia Comparison of the Model

We now provide an additional point of comparison to better understand the improvements from incorporating optimization into community-health program design. Even with the extension of the model seen in Appendix E, we are still comparing projections for programs that are optimized, with no quantified understanding of gains from applying this optimization

approach to the decision-making process. With this aim, we compare the program recommended by the model to the current structure of the CHW program in Ethiopia. Ethiopia is used as a point of comparison both for its geographical proximity and its status as a success story for community health programs in developing countries. As of 2015, an estimated 128,000 CHWs were operating in Ethiopia (Perry *et al.* 2014). From 2004, when the current version of the CHW program was introduced in Ethiopia, to 2020 the infant mortality rate fell from 73 per 1000 live births to 35 (World Bank 2020a). During the same time in Somalia, the infant mortality rate only fell from 104 to 73, a 52.05% decrease in Ethiopia with only a 29.81% decrease in Somalia (World Bank 2020b).

Because of the success in Ethiopia, a reasonable policy when establishing a community health program in Somalia would be to copy the interventions used in Ethiopia. We artificially replicate this approach by taking the list of interventions and their corresponding levels of coverage in Ethiopia, according to the LiST database, and applying them to the Somalia setting. In this way, we offer the same treatments in Somalia as are offered in Ethiopia. Since the CHW program in Ethiopia is much larger than the nascent program in Somalia, we cap the possible levels of coverage for the interventions with the FHW capacity (d). We then calculate the number of lives saved and the demand for the resources available. The coverage levels for this application are shown in Table 27.

Table 27: Ethiopia CHW intervention coverage levels applied to Somalia FHW program

Intervention	x_j: Coverage change (%)	Coverage target (%)	Current coverage (%)
Tetanus toxoid vaccination	9.88	76.88	67
MM supplementation in pregnancy		0	0
Iron supplementation in pregnancy	9.88	9.88	0
Calcium supplementation		0	0
Balanced energy supplementation		0	0
KMC - Kangaroo mother care		5	5
Breastfeeding promotion	9.88	18.88	9
Hand washing with soap		10	10
Prevention of malaria in pregnancy		2	2
Basic sanitation		38	38
Clean birth environment	9.88	16.88	7
Clean cord care	9.88	17.88	8
Immediate drying and stimulation	9.88	17.88	8
Neonatal resuscitation	9.88	13.88	4
Uterotonics for postpartum hemorrhage		7	7
Syphilis detection and treatment	9.88	13.88	4
Thermal regulation	9.88	17.88	9
Manual removal of placenta		3	3
Point-of-use filtered water		4	4
Malaria case management	9.88	10.88	1
Injectable antibiotics for neonatal sepsis	9.88	18.88	9
Safe abortion services		3	3
Oral antibiotics for neonatal sepsis		0	0
Post abortion case management		0	0
Antibiotics for treatment of dysentery	9.88	9.88	0

The package of interventions shown in Table 27 is projected to lead to 1,835 lives saved. This is 155 fewer lives saved than the optimized version in Table 19. Yet, it is achieved through the use of resources that far outstrip those available in Somalia at this time. In Table 28 we list the amount of each constraint used to achieve the levels of coverage offered in Table 27.

Table 28: Constraints on FHWs

Constraint	Budget	Usage
Total time available per month for care	22,602 hours	18,119 hours
Total supervisor time per month for training	3,927 hours	1,497 hours
Total budget available for scaling interventions	$ 435,000	$ 1,218,010
Total time available in curriculum	1,200 mins	2,225 mins

It is clear from Table 28 that by simply applying the interventions used in Ethiopia to Somalia, the projection of 1,835 lives saved is unrealistic, as the resources required in terms of money and curriculum time are greater than those available under our optimization projection. Furthermore, if we do relax the constraints to reflect the capacity implied by the Ethiopia levels of coverage (i.e. increase the budget to $1,218,010 and the curriculum time to 2,225 minutes), the optimized model achieves 2,417 projected lives saved in Somalia.

References

[1] Darmstadt, G.L., Bhutta, Z.A., Cousens, S., Adam, T., Walker, N. & de Bernis, L. (2005). Evidence-based, cost-effective interventions: how many newborn babies can we save? *Lancet, 365*, 977–988.

[2] Hawes, R.A., Thomas, A.L., Bhutta, Z.A., & Darmstadt, G.L. (2007). Impact of packaged interventions on neonatal health: a review of the evidence. *Health Policy and Planning, 22*(4), 193–215.

[3] Karsu, O. & Morton, A. (2021). Trading off health and financial protection benefits with multiobjective optimization. *Health Economics, 30*, 55–69.

[4] Marseille, E., Larson, B., Kazi, D.S., Kahn, J.G. & Rosen, S. (2015). Thresholds for the cost–effectiveness of interventions: alternative approaches. *Bulletin of the World Health Organization, 93*, 118–124.

[5] Nandi, A., Colson, A.R., Verma, A., Megiddo, I., Ashok, A., & Laxminarayan, R. (2016). Health and economic benefits of scaling up a home-based neonatal care package in rural India: a modelling analysis. *Health Policy and Planning, 31*(5), 634–644.

[6] Ochalek, J., Revill, P., Manthalu, G., McGuire, F., Nkhoma, D., Rollinger, A., Sculpher, M. & Claxton, K. (2018). Supporting the development of a health benefits package in Malawi. *BMJ Global Health, 3*.

[7] Ochalek, J., Lomas, J. & Claxton, K. (2019). Estimating health opportunity costs in low-income and middle-income countries: a novel approach and evidence from cross-country data. *BMJ Global Health, 3*.

[8] Qin, H., Xiao, J., Ge, D., Xin, L., Gao, J., He, S., Hu, H. & Carlsson, J.G. (2022). JD.com: Operations Research Algorithms Drive Intelligent Warehouse Robots to Work. *Informs Journal on Applied Analytics, 52*(1), 42–55.

[9] Stinnett, A.A. & Paltiel, A.D. (1996). Mathematical programming for the efficient allocation of health care resources. *Journal of Health Economics, 15*, 641–653.

[10] Stuart, R.M., Fraser-Hurt, N., Shubber, Z., Vu, L., Cheik, N., Kerr, C.C. & Wilson, D.P. (2023). How to do (or not to do)... health resource allocations using constrained mathematical optimization. *Health Policy and Planning, 38*(1), 122–128.

[11] van Baal, P., Morton, A. & Severens, J.L. (2018). Health care input constraints and cost effectiveness analysis decision rules. *Social Science & Medicine, 200*, 59–64.

[12] The World Bank. (2020a). *Mortality rate, infant (per 1,000 live births)- Ethiopia.* https://data.worldbank.org/indicator/SP.DYN.IMRT.IN?end=2020&locations=ET&start=1966&view=chart (26 March 2025)

[13] The World Bank. (2020b). *Mortality rate, infant (per 1,000 live births)- Somalia.* https://data.worldbank.org/indicator/SP.DYN.IMRT.IN?end=2020&locations=SO&start=1966&view=chart (26 March 2025)
